# Dyslexia and age related effects in the neurometabolites concentration in the visual and temporo-parietal cortex

**DOI:** 10.1038/s41598-019-41473-x

**Published:** 2019-03-25

**Authors:** Bartosz Kossowski, Katarzyna Chyl, Agnieszka Kacprzak, Piotr Bogorodzki, Katarzyna Jednoróg

**Affiliations:** 1 Faculty of Electronics and Information Technology Warsaw University of Technology, Nowowiejska 15/19, 00-665 Warsaw, Poland; 2 Laboratory of Brain Imaging, Nencki Institute of Experimental Biology of Polish Academy of Sciences, 3 Pasteur Str., 02-093 Warsaw, Poland; 3 Laboratory of Language Neurobiology, Nencki Institute of Experimental Biology of Polish Academy of Sciences, 3 Pasteur Str., 02-093 Warsaw, Poland; 4 Faculty of Psychology, University of Warsaw, Stawki 5/7, 00-183 Warsaw, Poland

## Abstract

Several etiological theories, in particular neuronal noise and impaired auditory sampling, predicted neurotransmission deficits in dyslexia. Neurometabolites also affect white matter microstructure, where abnormalities were previously reported in dyslexia. However findings from only few magnetic resonance spectroscopy studies using diverse age groups, different brain regions, data processing and reference scaling are inconsistent. We used MEGA-PRESS single-voxel spectroscopy in two ROIs: left temporo-parietal and occipital cortex in 36 adults and 52 children, where half in each group had dyslexia. Dyslexics, on average, had significantly lower total N-acetylaspartate (tNAA) than controls in the occipital cortex. Adults compared to children were characterized by higher choline and creatine in both areas, higher tNAA in left temporo-parietal and lower glutamate in the visual cortex, reflecting maturational changes in cortical microstructure and metabolism. Although the current findings do not support the proposed etiological theories of dyslexia, they show, for the first time, that tNAA, considered to be a neurochemical correlate of white matter integrity, is deficient in the visual cortex in both children and adults with dyslexia. They also point that several neurotransmitters, including ones previously used as reference, change with age.

## Introduction

Dyslexia is a brain-based difficulty in acquiring fluent reading skills that affects around 10–15% of the population^1^. Since this disorder is heterogeneous, many different etiological theories have been proposed to explain its symptoms^2^. Two were specifically linked to neurotransmission deficits, even though still little is known about neurochemical underpinnings of reading disorder. The first theory suggests that dyslexia might be a consequence of neuronal hyperexcitability, which contributes to learning deficits by heightened noise and instability in information processing^3^. This theory emphasizes the importance of balanced levels of excitation and inhibition within cortical pyramidal-interneuron networks, which support tuning to sensory input, neural timing, and information processing. Excitation–inhibition balance and the precise timing of neural activity can be disrupted by cortical hyperexcitability. On the level of neurotransmitters, this theory predicts heightened level of glutamatergic (Glu or Glx) signalling in dyslexic individuals^3^, since glutamate concentrations were found to be positively correlated with cortical excitability^4^. The loss of neuronal synchronization is hypothesized to lead to deficits in low-level temporal auditory processing, the oscillatory neural processes that sample and encode sensory information and impairments in multisensory integration, all of which are key components of reading development. Other theory of impaired auditory sampling^5^ suggests that the cause of reading disorder lies in a general deficit in synchronization of neuronal oscillations specifically in auditory cortex (phase-locking) in response to external input. However, studies examining cortical oscillations in dyslexia are inconclusive. Some point to synchronization deficits in the delta and theta range of 2–10 Hz^5^ which corresponds to syllable stress pattern, other show impairments in the low gamma band 25–35 Hz^6^, which corresponds to phonemic rate pattern. The two mentioned theories of dyslexia are not necessarily contradictory with each other, since the heightened noise level due to neural hyperexcitability might potentially lead to the imperfect synchronization of specific neuronal oscillations (phase-locking) in auditory cortex resulting in impaired auditory sampling.

On the level of neurotransmitters, gamma-aminobutyric acid (GABA) drives the modulation of gamma band^7^ and gamma band neurophysiological correlates of GABA concentrations have been observed in the visual^8,9^; temporal^10^ and motor^11^ cortex of healthy adults. The role of neurotransmitters in slow frequency theta oscillations is much less understood, but glutamate and choline (Cho) were implicated as the dominant neurotransmitters. Specifically during auditory signal processing in humans an association between glutamate concentration in hippocampus and theta oscillations in frontal areas was reported^12^. In rodents, hippocampal acetylcholine and theta oscillations seem to be tightly coupled as acetylcholine release accompanies the appearance of theta oscillations^13^ (for review see Pignatelli *et al*.^14^). Since the cortical oscillations studies in dyslexia did not provide conclusive evidence in favour of either theory, here we aim to directly test their predictions by examining if the concentrations of the above mentioned neurometabolites (i.e. GABA, choline and glutamate) are altered in individuals with dyslexia.

At the same time, studding alterations in neurometabolite levels may inform about underlying cytoarchitectonic differences between typical and dyslexic readers. It has been shown that total N-acetylaspartate (tNAA) concentration reflects neuronal density, function or viability^15^, but also maintains and supports myelination^16^. Choline is considered a marker of cell membranes in the voxel, which could reflect glial cell density^17^, the amount of myelin^18^ or the amount of membrane turnover from breakdown or synthesis^19^. Therefore increased choline was previously interpreted as reflecting either abnormal myelination or excessive cortical connectivity and thus longer communication route and transmission time^20^. In the studies where myelination was indirectly measured via fractional anisotropy (FA), significant differences were found between dyslexics and controls - both adults and children, especially in the arcuate fasciculus and corpus callosum^21^. Thus, various neurotransmitters can affect the neural structure and function at the macroscopic level with specific contributions to white matter microstructure. At the same time dysfunction in cortical connectivity has been hypothesized in individuals with dyslexia by multiple theories of dyslexia, not limiting to the hyperexcitability and impaired auditory sampling models. Overall, the findings of the neurometabolites in dyslexia can provide valuable insights to a broader understanding of neurobiological mechanisms underlying dyslexia.

Until now, only few studies investigated concentrations of neurometabolites in dyslexia. Initial work was carried out by Rae^22^, who found lower choline/N-acetylaspartate (NAA) ratio in the left temporo-parietal lobe and right cerebellum in 14 dyslexic compared to 15 typically reading males. Additionally, the researchers noticed significant lateralization differences in NAA in the dyslexic group, with lower concentration of NAA in the left compared to right hemisphere. Laycock *et al*.^20^ studied neurometabolites in the cerebellum and contradictory to Rae *et al*.^22^ found increased Cho/creatine and Cho/NAA in 6 dyslexic adults compared to 6 controls. Subsequently, on a bigger sample of 31 adults Bruno *et al*.^23^ revealed that in the left angular gyrus Cho/creatine ratio is negatively correlated with phonological awareness (PA), a skill closely related to reading^24^. Later, in a group of 75 children (6–10 years old), Pugh and colleagues^25^ showed a negative correlation of Cho/creatine in the occipital cortex with reading performance, but not with PA. They additionally showed negative correlations between Glu/creatine ratio and reading skill, as well as PA in the same occipital region. In this sample only 10 children met the criteria for reading disorder and when compared to 47 typical readers they showed elevated levels of both Cho and Glu. Pugh *et al*.^25^ was also the first to measure GABA concentration with PRESS edited sequence, however no significant effects in GABA concentration were observed. Recently, this group has published a follow up study^26^ where they reanalyzed spectroscopy from a subsample of 70 children from 2014^25^. Additionally to previous work they reported correlations with response time in cross-modal word matching task where lower GABA/Cr and higher NAA/Cr concentration predicted faster reaction times. What is more, authors showed that the task response time mediates between metabolite concentration (Glu, Cho) and reading ability. Although, main result concerns GABA and NAA differences in cross-modal matching, the mediation in Glu and Cho is highlighted as supportive argument for neural noise hypothesis. Finally, Lebel *et al*.^27^ measured neurometabolites’ concentration in the anterior cingulate gyrus (n = 56) and left angular gyrus (n = 45) in a group of preschool children. They reported positive correlations between PA and neurotransmitters - Glu, creatine and inositol in anterior cingulate. Positive correlation of PA and Glu reported by Lebel *et al*.^27^ is in the opposite direction to the findings of Pugh *et al*.^25^. Similarly Rae *et al*.^22^ found decreased, while others^20,25^ increased concentration of choline in dyslexic compared to control samples.

Among the potential reasons causing the inconsistencies in previous studies might be the differences in the age of participants (pre-reading children vs. school-age children vs. adults), differences in the reference scaling (absolute concentration vs. reference to creatine or NAA) as well as brain regions examined (left temporo-parietal vs. occipital vs. anterior cingulate cortex vs. cerebellum). Additionally, even though in each previous study several different neurometabolites were examined, the authors used a nominal significance of p < 0.05 to report positive findings of group differences^20,22,25^, which might have led to false positives. Neurometabolites concentration is likely to differ between age ranges, reflecting changes in glutamatergic neurotransmission and refinement of cortical networks in particular through axonal elongation, myelination and pruning^28–30^. It has been shown that during initial education (5–12 years) NAA rises by 7% and Cr by 10% in occipital and parietal voxels respectively^29^. The same authors did not obtain significant results in mid education group (12–18 years). In a wider age group (4–13 and 18–33 year olds analyzed together) negative slope of Glu/Cr ratio was determined^31^ with authors’ assumption that Cr concentration remains stationary. In fact, when absolute concentrations of Glu and Cr were analyzed^30^, both were characterized with negative age slope in participants aged from 18 to 31. Bearing in mind these maturational changes (in particular glutamate decreases^31^ and NAA rises^29^ from childhood to young adulthood in occipital cortices), it is difficult to form definite conclusions about the alternations of brain neurochemistry in dyslexia. Furthermore, so far only three studies directly compared dyslexic to typically reading subjects^20,22,25^, but all had relatively small samples of dyslexic subjects.

Here we aimed at systematic analysis of potential neurometabolite abnormalities in dyslexia by examining the concentration of glutamate (Glu and Glx), choline, NAA and GABA in both adult and children samples. Additionally, we tested whether Cr level can be utilized as a reference. We hypothesize that, if reading disorder is associated with poor phase synchronization in low frequency bands, as suggested by Goswami^5^, dyslexic groups should show abnormalities in the concentration of glutamate and/or choline. If, however, as suggested by Lehongre *et al*.^6^, dyslexia is related to poor synchronization in the gamma band, concentration of GABA should be altered. Heightened glutamate in dyslexics compared to control subjects is also expected by the neuronal noise theory^3^. Finally, changes in NAA and choline can inform about white matter microstructure differences previously implicated in dyslexia^21^. Since still little is known about whether dyslexia effects change with age, besides the effect of group (dyslexic vs. typical readers), we examined both age effects and interactions on neurometabolites’ concentration. Thus the present study, as compared to previous ones examining similar age ranges^23,25,27^, has an additional insight of examining the commonalities and differences between school-aged children and experienced adult readers in the level of neurotransmiters in two brain regions present in the previous studies (i.e. occipital cortex, and left temporo-parietal cortex including the angular gyrus). By examining two different brain regions in the same subjects we can infer about the specificity of neurotransmiter differences in dyslexia. Finally, in the supplementary analyses we compare different reference scaling methods and suggest directions for future research. In order for the reader to compare current findings with the previous literature we report all effects surviving the nominal significance of p < 0.05, but focus more on results surviving more stringent statistical threshold corrected for multiple comparisons.

## Results

### Behavioral measures

In adults Adult Reading History Questionnaire(ARHQ) score (40.6 ± 17.9) was highly correlated with word reading (r = −0.57, p < 0.001) and pseudoword reading (r = −0.73, p < 0.001). In both reading tests adults with dyslexia performed significantly lower than adult control group (p < 0.001, see Table 1). Dyslexic adults were also significantly slower than controls at naming letters and numbers in Rapid automatized naming (RAN), but had similar performance in the objects and colors subtests. This pattern of results remained unchanged when several subjects were removed from the analysis due to spectral artifacts. It is important to mention that both ARHQ and pseudowords reading test were used to classify subjects to the groups in adult sample.

**Table 1:** Adult and children group characteristics.

Characteristic	Adults				Children			
	Dyslexic N = 18	Control N = 18	t|χ2	p	Dyslexic N = 26	Control N = 26	t|χ^2^	p
Age	30.28 ± 4.09	28.02 ± 3.40	1.804 (34)	0.080	10.90 ± 0.98	11.21 ± 0.95	1.136 (50)	0.261
Male	13 (72.2%)	11 (61.1%)	0.500 (1)	0.480	15 (57.7%)	15 (57.7%)	0.000 (1)	1.000
ARHQ	55.78 ± 7.18	25.39 ± 11.11	9.772	<0.001	—	—	—	—
Words/min	115.67 ± 12.45	142.50 ± 16.74	−5.456	<0.001	47.62 ± 16.61	95.5 ± 20.11	−9.361	<0.001
Pseudowords/min	62.28 ± 10.39	90.44 ± 15.33	−6.456	<0.001	33.27 ± 6.55	57.92 ± 14.74	−7.794	<0.001
RAN (objects and colors) in sec	62.61 ± 7.35	59.11 ± 8.98	1.280	0.209	95.88 ± 15.71	80.65 ± 11.86	3.947	<0.001
RAN (letters and numbers) in sec	37.44 ± 4.13	32.72 ± 5.88	2.788	0.009	57.04 ± 10.49	47.92 ± 9.41	3.298	0.002
Phonological awareness (sten)					3.50 ± 1.70	5.46 ± 2.02	3.781	<0.001

Dyslexic children had significantly worse performance than children from the control group in all behavioral tests - word and pseudoword reading, both subscales of RAN (objects & colors and letters & digits), and phonological task based on pseudowords. This pattern of results was not affected by removing subjects with spectral artifacts. Descriptive statistics and results of the statistical comparisons between the dyslexic and control groups are presented in the Table 1.

### Spectroscopy - data quantity and quality

In adult group 4 spectra from visual cortex and 3 from left temporo-parietal cortex were rejected from the analysis due to strong artifacts. Two additional GABA estimates from visual cortex were rejected after GANNET preprocessing. In the end, 33 spectra from the left temporo-parietal lobe (17 dyslexic and 16 control adults) and 32 spectra from the visual cortex (16 dyslexic and 16 control adults) were being taken under consideration (30 GABA estimates from visual cortex − 15 dyslexic and 15 control adults).

In children sample, after data reconstruction 1 subject was rejected due to heavy, presumably motion artifacts in both spectra. Eight more spectra from left temporo-parietal cortex and 1 from visual cortex were declassified upon detailed visual inspection of LCModel printout. Additionally, 4 GABA estimates were rejected after preprocessing in GANNET (3 from left temporo-parietal cortex and 1 from visual cortex). Invalid spectra were misaligned during spectral registration and subsequently characterized with an abnormal shape of peaks and baseline. As a result, 43 spectra from left temporo-parietal lobe (20 dyslexic and 23 control children) and 50 spectra from visual cortex (25 dyslexic and 25 control children) were further analyzed (40 − 17 dyslexic and 23 control children and 49 − 25 dyslexic and 25 control children GABA estimates, respectively).

### Spectroscopy - group analyses

#### Choline (Cho)

Two way ANOVA revealed a significant main effect of age in both left temporo-parietal cortex and visual cortex (F(1,72) = 30.68, p < 0.001***, BF_10_ > 1000 and F(1,78) = 30.19, p < 0.001***, BF_10_ > 1000 respectively). Adults on average had higher choline concentration than children by 14.1% in temporo-parietal and 10.1% in visual cortex voxels. Interactions between age and group reached nominal significance, but did not withstood correction for multiple comparisons with F(1,72) = 5.15, p = 0.026, BF_10_ = 2.459 (left temporo-parietal cortex) and F(1,78) = 6.52, p = 0.013, BF_10_ = 4.007 (visual cortex). Dyslexic children compared to control children had lower by 7.6% absolute choline concentration in the left temporo-parietal (p = 0.05, BF_10_ = 1.438) and by 5.5% in visual cortex (p = 0.031, BF_10_ = 1.466) (uncorrected for multiple comparisons). In adults the difference between the experimental groups did not reach nominal significance in neither regions, although dyslexic adults tended to have higher choline concentration than controls. Therefore there was no main effect of dyslexia.

#### Glutamate and glutamine (Glu & Glx)

In the visual, but not in the left temporo-parietal cortex, a main effect of age was found for both Glu and Glx (F(1,78) = 52.03, p < 0.001***, BF_10_ > 1000 and F(1,78) = 33.53, p < 0.001***, BF_10_ > 1000 respectively), with children having higher concentration of neurotransmitters than adults (17.9% - Glu and 13.6% - Glx). There was no significant effect of group nor interaction between age and group in neither Glu nor Glx.

#### Gamma-aminobutyric acid (GABA)

In both brain areas, neither main effects of age and group nor interaction between age and group reached significance for GABA.

#### Creatine (Cr)

In both brain areas there was a significant effect of age (F(1,72) = 42.79, p < 0.001***, BF_10_ > 1000 for the left temporo-parietal and F(1,78) = 12.22, p = 0.001**, BF_10_ = 37.307 for visual cortex). Children had lower concentration of Cr than adults by 17.8% in temporo-parietal and by 5.7% in the visual cortex.

#### Total N-acetyl-aspartate (tNAA)

**Figure 1:**
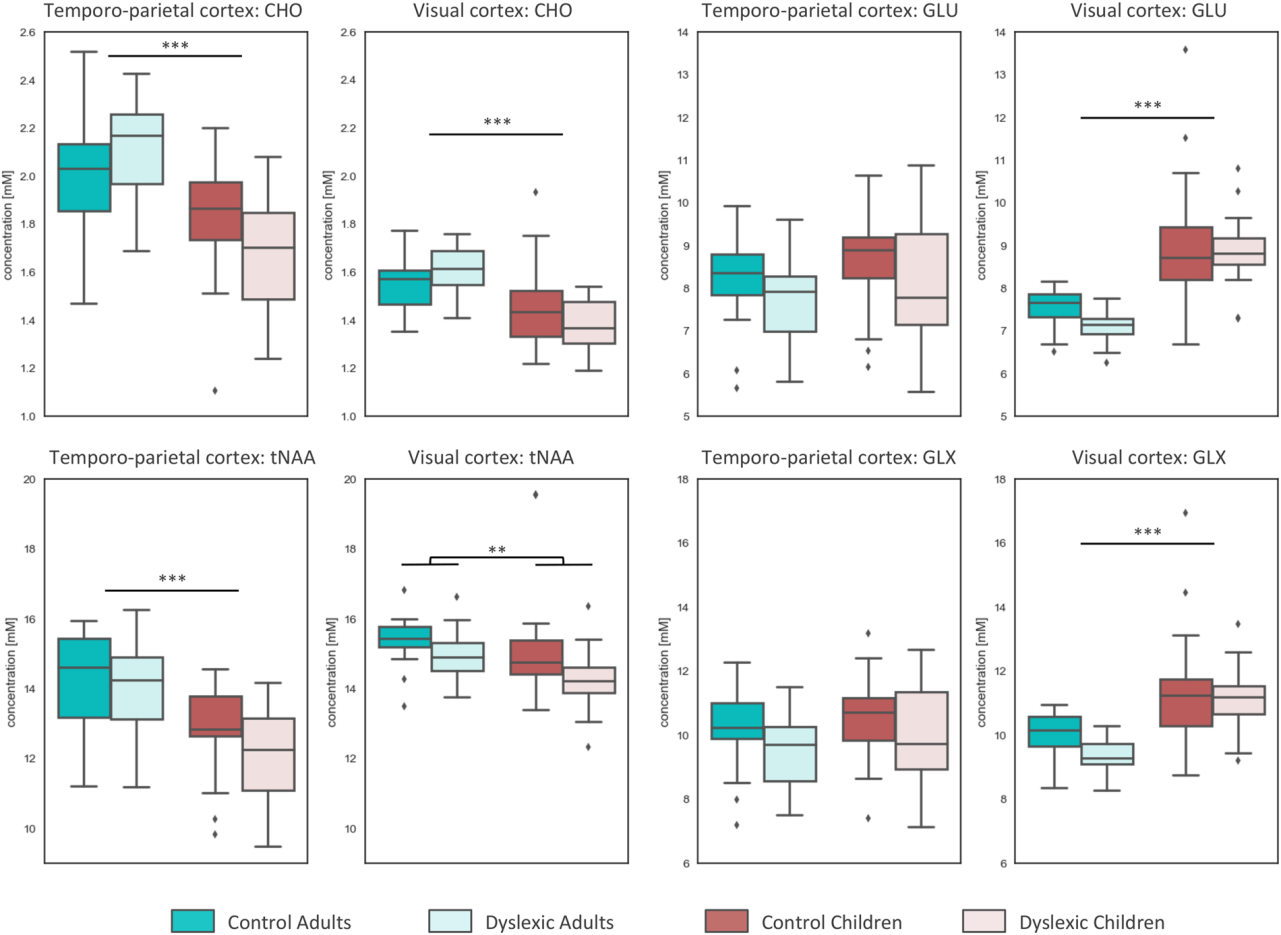
The concentration of choline (Cho), N-acetyl-aspartate (tNAA), glutamate (Glu & Glx) in control and dyslexic adults and children in the left temporo-parietal and occipital cortex. Significant effect of age is present in the left temporo-parietal cortex for Cho and tNAA, and in the visual cortex for Cho and glutamate (Glu & Glx). Significant effect of group is observed for the tNAA concentration in the visual cortex. ***p < 0.001, **p < 0.01.

In the visual cortex, but not in the left temporo-parietal cortex, there was a significant main effect of dyslexia for the tNAA concentration (F(1,78) = 7.52, p = 0.008**, BF_10_ = 9.811, see Fig. 1). The effect remained significant (F(1,76) = 7.23, p = 0.009**, BF_10_ = 4.598) after removal of two control children with outlying tNAA concentration (>3 SD above mean). Subjects with dyslexia had on average lower concentration of tNAA by 4.5% than typically reading controls. There was also a significant effect of age in the left temporo-parietal cortex (F(1,72) = 23.73, p < 0.001***, BF_10_ > 1000), where children had lower concentration of tNAA than adults by 10.8%.

#### Brain - behavior correlations

**Figure 2:**
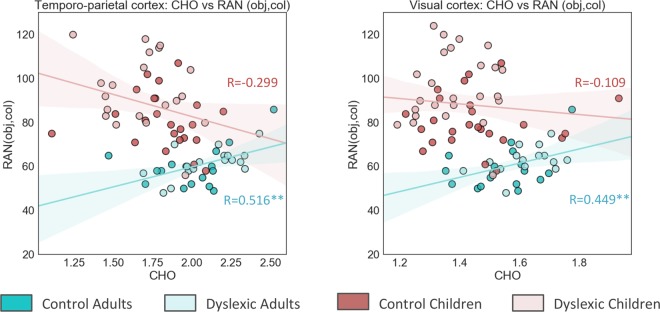
Correlations between neurometabolites and behavioral tests in adults and children separately. **p < 0.01.

We performed correlations within the group of adults and children, combining both control and dyslexic individuals. This analysis showed that choline concentration correlated with RAN objects and colors differently in adults and children. The correlation was positive in adult sample (r = 0.516, p = 0.002** in the left temporo-parietal cortex and r = 0.449, p = 0.01 in the visual cortex), meaning the higher the concentration of this metabolite the slower naming speed (see Fig. 2). In children the correlation between RAN and choline was of a different direction, i.e. the higher the concentration of choline the faster naming speed, but it did not approach significance (r = −0.299, p = 0.052 in the left temporo-parietal cortex and r = −0.109, p = 0.45 in the visual cortex). Importantly, the difference in correlations coefficients between adults and children was significant (z = 3.64, p < 0.001 for the temporo-parietal and z = 2.51, p = 0.012 for visual cortex). Several weaker correlations were found, but they did not survive the correction for multiple comparisons. Specifically, in children there was a positive correlation between choline concentration in the visual cortex and words reading (r = 0.284, p = 0.045) and a negative correlation between tNAA in the temporo-parietal cortex and time needed to name objects and colors (r = −0.372, p = 0.014). Phonological awareness in children was also correlated with metabolite concentration, but no significant effects were found. In adults, glutamate in the left temporo-parietal cortex was positively correlated with RAN objects and colors (r = 0.367, p = 0.036 - Glu; r = 0.385, p = 0.027 - Glx).

## Discussion

The current study is the first to examine brain neurochemistry in typically developing and dyslexic children and adults in the two brain regions: occipital cortex, and left temporo-parietal cortex including the angular gyrus. We tested the predictions of different etiological theories of dyslexia related to neurotransmission deficits and aimed to disentangle inconsistencies between previous findings by exploring how participants’ age or different preprocessing steps (see supplementary materials) affect the results.

We found that dyslexic individuals, irrespective of age, had significantly lower tNAA than controls in the occipital cortex. This was the only group effect related to dyslexia, which survived the correction for multiple comparisons. NAA is the most prominent neurotransmitter detected in the normal human brain, which reflects neuronal density, function or viability^15^, but is also considered to be a neurochemical correlate of neuron-oligodendrocyte (axon-myelin) integrity^32^. Reduced tNAA in dyslexic subjects suggests white matter microstructure abnormalities since the concentration of tNAA is positively correlated with fractional anisotropy (FA)^33,34^. FA measures the amount of coherence of water diffusion which putatively reflects the amount of myelination in axonal bundles or the coherence of fiber tracts. Diffusion weighted studies identified lower FA mainly in the left temporo-parietal areas in dyslexia in both children and adults^21^, whereas occipital areas seem to be less frequently found^35,36^. However, occipital cortex was for a long time a focus of neurofunctional studies on dyslexia, showing the abnormalities in processing of visual motion^37^ and other stimuli such as symbol strings^38^. NAA differences in reading skill were identified only in one recent MRS study, where higher NAA/Cr concentration in the occipital cortex predicted faster reaction times in a cross-modal matching task with language stimuli (letters, words and pseudowords) in children^26^. This result would be in line with ours, where controls had higher concentration of tNAA in the occipital cortex than dyslexics. Del Tufo and colleagues suggested that higher NAA in the occipital cortex corresponds to more intact white matter reading network and specifically to left arcuate fasciculus in the temporo-parietal cortex, which connectivity was related to individual differences in cross-modal brain activity in developing readers. However, we found no effects of dyslexia in the concentration of tNAA in the left temporo-parietal cortex. Moreover, lower tNAA in the occipital cortex characteristic for dyslexic subjects in the current study was also not predicted by the two etiological theories of dyslexia.

Neuronal noise hypothesis predicted heightened level of glutamate^3^ in dyslexia. However no such effect was present in the current study. So far only one study reported elevated levels of Glu/creatine in dyslexic children in the occipital cortex^25^. Even though the stated effect size for this group difference was large (Cohen’s *d* = 0.79), given the low sample of dyslexic children (n = 10) and no correction for multiple comparisons it is not unlikely that this estimate might be inflated^39^. Previous correlational MRS studies showed mixed results for glutamate, with positive correlation with phonological awareness in anterior cingulate in pre-reading children^27^ and negative correlation for Glu/creatine with both reading performance and phonological awareness in the occipital cortex in school-age children^25^. In our study, glutamate concentration was not related to reading, phonological awareness or naming speed in children, whereas in adults higher glutamate concentration was related to slower naming speed, but this result did not survive correction for multiple comparisons.

Altered levels of glutamate together with choline would be also expected by impaired auditory sampling theory for theta oscillations, which correspond to speech syllables^12,14^. With respect to choline, our results suggest that its role in reading and dyslexia can change with age. Specifically, in adults, but not in children, higher choline in both occipital and temporo-parietal cortex was related with slower naming speed in the RAN test. This result is in line with Bruno *et al*. (2013), who found a negative correlation between choline in left angular gyrus and phonological skills in young adults^23^. RAN is considered to be a phonological skill reflecting the speed of access to phonological information in the long-term memory^40^. Similarly to PA, RAN is a reliable predictor of reading skills, especially in transparent orthographies such as Polish. In contrast to Pugh *et al*. (2014), where a negative correlation between Cho/creatine and reading skills was found in occipital cortex in children, we did not observe any significant correlations between choline and reading or reading related skills in children. Here, we found that the concentration of creatine might change with age, as adults had higher absolute concentration of creatine than children. Given this, pairwise correlations between Cho/creatine and reading skills not adjusted for age might be difficult to interpret^25^. Additionally, we found anecdotal evidence for an interaction between age and group in choline concentration both in temporo-parietal and occipital cortex with dyslexic children compared to control children having lower choline concentration (nominally significant), whereas dyslexic adults having higher choline concentration than typically reading adults. Importantly, such interaction was observed irrespective of scaling or correction for tissue composition (see Supplementary Materials). Future studies should test if this result holds in larger samples. Our results are not consistent with Rae’s *et al*.^22^ study, where Cho/NAA ratio was reported to be lower in temporo-parietal regions in adult dyslexic men compared to controls (but see Laycock *et al*. 2008 for findings in the opposite direction in the cerebellum). The discrepancy might be related to reference scaling to NAA, when they used scaling to creatine differences between groups became non-significant^22^. In fact, in the current study the absolute concentration of tNAA differed between dyslexic and control subjects in the visual cortex and between children and adults in the temporo-parietal cortex, yielding scaling to tNAA illegitimate. Our results stand also in contrast to previous findings of elevated levels of Cho/creatine in dyslexic children in the occipital cortex^25^. Since the results of both their and our study survived only nominal significance, future research in larger samples is needed to resolve these discrepant findings.

Meanwhile, no significant differences in GABA concentration, which drive oscillations in high frequency bands, were found in neither of the groups. GABA is still difficult to measure compared to glutamate or choline concentration and even with an improved data processing pipeline we did not come to conclusive results. The calculated Bayesian factor (alternative/null) suggested that the data were 0.296:1 for visual and 0.427:1 for temporo-parietal in favor of the null hypothesis (no differences between controls and dyslexic), or rather, 3.38 or 2.34 times more likely to occur under a model without including an effect of dyslexia, rather than a model with it. Alternatively, it would take more than 272 subjects to prove 2% difference in GABA concentration in the left temporo-parietal cortex based on power analysis. Thus GABA (if relevant) needs to be further examined in a much larger sample with a more robust protocol.

Apart from the effect of dyslexia on the concentration of neurometabolites, we observed several maturational changes. In particular adults compared to children were characterized by lower glutamate, especially in the visual cortex, in line with previous studies^30,31^. Age-related reduction in density of glutamatergic NMDA receptors^41^ and glutamate-glutamine cycle flux^42^ could lead to reduced glutamate driven activation and cognitive deficits. However, since glutamate reduction was related to age but not dyslexia in our sample, the decrease in metabolites is rather the result of maturation of neuronal system and its metabolism. Such an effect, reviewed by Segovia in 2001, is linked to elevated metabolic activity in children and changes in glutamatergic neurotransmission^28^. What is new in the field, higher choline levels in adults than in children were found in both visual and temporo-parietal cortex. As stated in introduction, choline is considered a marker of cell membranes in the voxel i.e. the amount of membrane turnover from breakdown or synthesis. It has been shown that choline pool measured in 1H-MRS and i.e. glycerophosphorylethanolamine and glycerophosphorylcholine measured by 31P-MRS increases with age^18^. Our results are consistent with age-related dynamics of tissue growth measured by MR relaxometry and diffusion^43^. The relation between visible choline and maturation was not well studied in humans but other authors reported increases in Cho related to aging in rats^44^. According to the their hypothesis the uptake of acetylocholine might be impaired by aging, leading to higher signal of free choline measured by MRS. The result presented here is especially interesting in the context of previously reported discrepancies in choline findings^20,22,23,25^ depending presumably on age of participants (anecdotal interaction was detected here). We also found that children had lower concentration than adults of tNAA in the left temporo-parietal cortex and creatine in both brain areas in line with increase measured in 5–12 y/o children^29^. To sum up, we observed increase in tNAA, Cr, Cho considered as markers of changes in cortical microstructure and Glu, a measure of increased metabolism. Thus, these results question the use of tNAA or Cr (generally thought to be stable^45^) as a reference when comparing different age groups i.e. in dyslexia studies. It seems that the normalization of metabolites to creatine or tNAA, limits possible interpretations and future studies measuring absolute metabolite concentrations relative to water signal would be more valuable.

A potential limitation of our study is that we used slightly different acquisition parameters in the group of children - shorter acquisition to reduce number of spectra affected by motion and smaller voxel in temporo-parietal lobe to better correspond with anatomical features. However, the applied corrections for partial volume effect and locally measured water signal make the spectra comparable between the groups with a use of applied statistical tests (assuming unequal variance where relevant). Nevertheless, measuring metabolites of low signal such as GABA is still a challenge in a reasonable amount of acquisition time. We look forward to novel methods of online motion correction for longer spectroscopic acquisition.

To conclude, although the current findings do not lend support to neither of the proposed etiological theories of dyslexia, they show, for the first time, that tNAA, considered to be a neurochemical correlate of white matter integrity, is deficient in the visual cortex in both children and adults with dyslexia. They also point that several neurotransmitters, including ones previously used as reference, change with age. However, current results should be interpreted with caution given our cross-sectional data. Future studies should employ a longitudinal approach to examine developmental changes in neurometabolites ideally from the pre-reading period.

## Methods

The general inclusion criteria for both adult and children samples were: good overall health, right-handedness, normal (or corrected to normal) vision, normal hearing, no history of neurological illness or brain damage and no symptoms of ADHD. All participants speak Polish as their first language. The study was approved by the Warsaw University Ethical Committee and carried out in accordance to the provisions of the World Medical Association Declaration of Helsinki. All adult participants and parents gave written informed consent to the study, and the children agreed orally.

### Participants - adults

36 adult volunteers (12 females/24 males, mean age 27.3 ± 3.8 y/o, 21.1–39.8 y/o) were included in the study. All participants completed higher education.To select subjects for dyslexic and control group, we used three different criteria and the subject had to meet at least two of them to be included in the dyslexic group. First criterion was the formal dyslexia diagnosis. However, even though 14 subjects obtained dyslexia diagnosis in childhood, dyslexia was not as commonly known and diagnosed in Poland in the 1980s. We thus decided to introduce additional criteria. The second one based on the ARHQ score, commonly used to screen for the reading problem in families in the dyslexia research. A criterion was met if the ARHQ score was higher than 40^46^. Third criterion was the pseudoword reading score. The criterion was met if the subjects obtained a score lower than group median (Med = 69, Mean = 76). 4 subjects that were not previously diagnosed with dyslexia met both criteria: for ARHQ and pseudoword reading. These subjects were also classified as dyslexics. All 14 individuals with dyslexia diagnosis in childhood also scored above 40 point cut off in the ARHQ. Then from the larger group of volunteers we selected subjects without problems with reading (subjects that met at most one of the criteria described above) matching for age and sex to the dyslexic group (see Table 1). In result, we selected 18 dyslexics and 18 controls for the analysis reported in the paper.

### Participants - children

52 children (22 females/30 males, mean age 11.06 ± 0.97 y/o, range 9–12.9 y/o) from third to fifth grade of elementary school were recruited from the larger sample taking part in a different study at the Nencki Institute. The dyslexia diagnosis^47^ and the IQ assessment (Wechsler Intelligence Scale for Children^48^) was conducted within this larger study. We selected 26 children with dyslexia diagnosis, and matched (by age and gender) 26 typically reading controls. All children had average or above-average IQ. The two groups did not differ in age or sex (see Table 1). Additionally, we made sure that the children were born at term (≥37 weeks).

### Behavioral measures

Apart from neuroimaging all subjects were tested on words and pseudowords reading speed^49^ as well as rapid automatized naming - RAN^50^ with two subtests: objects & colors and letters & digits. The amount of words and pseudowords read per minute was compared between the groups. For the RAN test, the summed time for naming objects and colors as well as letters and digits subtests was compared. All children were tested with a battery of phonological awareness tasks based on pseudowords in which both syllable and phoneme manipulation level was included^47^. Results of behavioral measures are presented in the Table 1.

### MR Spectroscopy and Imaging

**Figure 3:**
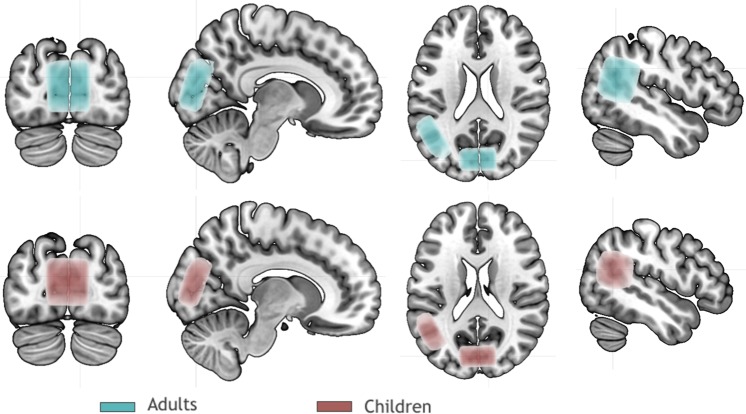
Mean position of all voxels after normalization to MNI space. Voxel mask was thresholded at 10% of relative intensity.

MRI was performed on Siemens Magnetom TRIO 3 T using 32CH receiving coil. MEGA-PRESS spectroscopy^51^ (Siemens research agreement WIP-529) was acquired from two locations – occipital (visual) cortex and left temporo-parietal cortex. Voxels were carefully placed based on 3D T1-weighted images (TR = 3000 ms, TI = 900 ms, 1 × 1 × 1 mm resolution) reconstructed in 3 planes (see Fig. 3).

Spectroscopy acquisition was done with TR = 2000 ms and TE = 68 ms. Acquisition protocol was validated in the group of adults with 192 averages for both edited and non-edited spectra (NA = 192*2). A special attention was paid to quality of MEGA-PRESS edited spectrum thus longer acquisition time was used to better estimate differential spectrum of GABA. However, spectra averaged across >10 min were frequently corrupted due to bad initial calibration, field drift or subjects’ motion. Further, we decided to shorten the acquisition in the whole group of children to NA = 96*2 (from 12 m 50 s to 6 m 25 s) and repeat whole measurement in case of strong artifacts (with the subject’s consent). We think that such modification was legitimate, since all metabolites of interest apart from GABA still withstood conservative threshold of CRLB < 15%^52^ (Cramér–Rao lower bound - an error of signal modeling used for estimating maximum variance in metabolites concentration). The acquisition parameters were comparable to recent studies where standard PRESS sequence was used^23,27^. In adults the voxel size was 30 × 30 × 15 mm in both the visual cortex and left temporo-parietal cortex. In children the voxel was of the same size in visual cortex while the size was slightly reduced in temporo-parietal lobe (from 30 × 30 × 15 to 25 × 25 × 15 mm) to better correspond with angular gyrus anatomical features.

Water unsuppressed scan (NA = 16) was acquired to reference the metabolites concentrations. T1-weighted volumes were segmented in SPM8 (New Segment) and coregistered with individual voxel masks in order to correct for partial volume effects. Spectral analysis was performed in Gannet^53^ - GABA and LCModel^53^ - alanine, aspartate, creatine, GABA, Glu, Gln, GSH, choline compounds (Cho), lactate, myo-inositol, NAA, NAAG, scyllo-inositol, and taurine. Spectra were assessed by a spectroscopist with a regard to SNR, shape of baseline, residual noise, symmetry and width of singlets (NAA, Cr) and voxel position. GABA estimation was performed in Gannet with the raw data (Siemens TWIX) with the following steps: (1) coil channel combine and phase correction, (2) frequency drift correction, (3) calculation of differential spectrum, (4) fitting with the gaussian peak. (5) Then, the results of GABA were corrected for partial volume effects in respect to CSF, GM/WM ratio as well as tissue specific relaxation times^54^. Some spectra were misaligned during GANNET preprocessing and also disqualified from analysis.

**Figure 4:**
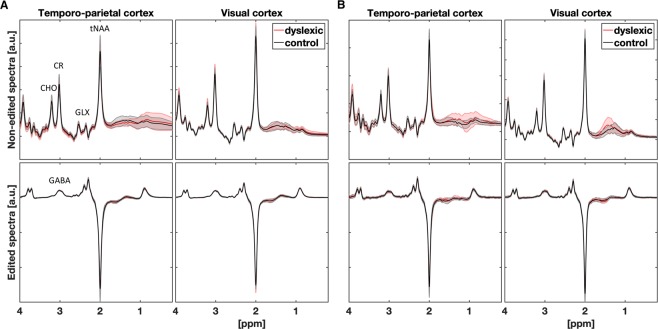
Mean spectra and standard deviation in (**A**) adult and (**B**) children groups.

LCModel was used to calculate absolute metabolite concentration based on MEGA-OFF spectrum (Fig. 4). Siemens RDA (spectra averaged on the scanner) were fitted with the simulated basis set consisting of 14 metabolites^55^. LCModel’s reference water concentration was corrected based on properties of voxel specific tissue volume fractions^56^.

### Metabolites of interest and confounding factors

According to presented literature we hypothesised that Cho, Glu (and Glx), NAA or GABA concentration would differ in dyslexia. Additionally, we tested whether Cr level can be utilized as a reference.

### Type of analysis, multiple comparison correction

We used a 2 × 2 ANOVA model with group (dyslexic vs. control) and age (adult vs. children) as factors. Post-hoc pairwise comparisons were performed if main effects or interaction were found significant. In order to compare current findings with the previous literature^20,22,25^ we report all effects surviving the nominal significance of p < 0.05, but focus more on results surviving more stringent statistical threshold corrected for multiple comparisons (5 metabolites) resulting in nominal threshold of p < 0.01. The results that survive correction for multiple comparisons are marked with 2 or more asterisks. Bayesian ANOVA was performed for estimating ratio of the likelihood probability of two competing hypotheses - Bayes factor^57^. Here we assume that for BF_10_ > 3 there is a substantial evidence for alternative hypothesis comparing to null and symmetrically for BF_10_ < 1/3 for null comparing to alternative. Bayes factor does not require multiple comparison correction^58^.

### Applied corrections and confounding factors

Different scaling methods may potentially lead to bias results i.e. when considering age and voxel position. We decided to show fully corrected concentrations for relaxation, tissue composition and scaled with water concentration within a voxel as a main result. Additionally, we were obliged to test for any effect of scaling methods used previously by referenced authors such as: scaling to tNAA or Cr and less sophisticated tissue correction. Such information is presented in supplementary materials for the purpose of comparisons with published studies and thus was not taken into account in multiple comparison correction.

### Brain behavior correlations

Furthermore, similarly to previous studies^23,25^ we performed Pearson’s correlation between Cho, Glu, Glx, GABA, tNAA and behavioral tests (words and pseudowords reading and RAN). The correlations were performed separately for adults and children.

## Supplementary information

Supplementary analyses

## Data Availability

The datasets generated and analyzed during the current study are available from the corresponding authors on request.
