# Supplementary material for: Dyslexia and age related effects in the neurometabolites concentration in the visual and temporo-parietal cortex
Source: Sci Rep. 2019 Mar 25;9:5096. doi: 10.1038/s41598-019-41473-x (PMC6434036; doi:10.1038/s41598-019-41473-x)
Supplement: Supplementary file 1 — Supplementary analyses [file 41598_2019_41473_MOESM1_ESM.docx]

**Dyslexia and age related effects in the neurometabolites concentration in the visual and temporo-parietal cortex**

**Bartosz Kossowski^1,2^***

b.kossowski@nencki.gov.pl

Phone: +48 22 5892 551;

**Katarzyna Chyl^3^**

**Agnieszka Kacprzak^3,4^**

**Piotr Bogorodzki^1^**

**Katarzyna Jednoróg^3^***

[k.jednorog@nencki.gov.pl](mailto:k.jednorog@nencki.gov.pl)

Phone: +48 22 5892 392; Fax: +48 22 5892 331

1. Faculty of Electronics and Information Technology

Warsaw University of Technology, Nowowiejska 15/19, 00-665 Warsaw, Poland

2. Laboratory of Brain Imaging

Nencki Institute of Experimental Biology, Polish Academy of Sciences

Pasteur 3, 02-093 Warsaw, Poland

3. Neurobiology of Language Laboratory

Nencki Institute of Experimental Biology, Polish Academy of Sciences,

Pasteur 3, 02-093 Warsaw, Poland

4. Faculty of Psychology

University of Warsaw,

Stawki 5/7, 00-183 Warsaw, Poland

* corresponding authors

**Supplementary materials**

Supplementary group analyses

Since previous studies did not correct for tissue composition in the voxel or reported additional values corrected only for linear effects of GMV within a statistical model[^18^](https://paperpile.com/c/GSpb2g/6PxFf), we performed supplementary group analyses where water referenced concentration of metabolites was analysed without correction for tissue volume fractions or with a control of variance explained by GMV by the means of multiple regression. This approach is different than the one presented in main body in such a way that full correction utilized percentage of 3 different tissue classes and respective relaxation times [^50^](https://paperpile.com/c/GSpb2g/FHike) in contrast to including gray matter as a covariate. Supplementary group analyses were also performed with creatine as an internal reference, again without correction for volume fractions or with variance partially explained by GMV since all previous studies besides one [^20^](https://paperpile.com/c/GSpb2g/4r5mo), used creatine concentration as a reference for other metabolites. Additional tests were not corrected for multiple comparisons. The results are presented in Table S1 for left temporo-parietal and Table S2 for occipital cortex.

Choline

In both temporo-parietal and visual cortex, the interaction between age and group reached nominal significance irrespective of scaling or correction methods (see Tables S1 and S2). In the temporo-parietal cortex, congruently with the main analysis (corrected for tissue composition), control children had higher choline concentration than dyslexic children, while the difference in adults, although going in a different direction, did not reach significance. In the visual cortex, the difference in adults was significant with higher choline concentration in dyslexic than control adults, while the difference in children with higher concentration in control than dyslexic (as in the main analysis) did not reach significance. Adults on average had higher concentration of choline than children and the age effect was more stable in the temporo-parietal cortex than in the visual cortex.

Glutamate and glutamine (Glu & Glx)

In the visual cortex there was a stable effect of age, with children having higher Glu and Glx concentrations than adults. In the temporo-parietal cortex this effect, although present, was less stable. Scaling to creatine, instead of water, irrespective of GMV correction resulted in a group effect in the temporo-parietal cortex, with lower Glu/creatine and Glx/creatine in dyslexic than control individuals.

Gamma-aminobutyric acid (GABA)

No significant effects for GABA were observed in the visual cortex. Scaling to creatine, instead of water, irrespective of GMV correction resulted in the main effect of age in the temporo-parietal cortex, with higher GABA/creatine concentration in children than adults.

Total N-acetyl-aspartate (tNAA)

The effects of age and group changed a lot depending on scaling and correction. Scaling to creatine, reversed age effect in the temporo-parietal cortex with children having higher concentration of tNAA/creatine than adults (adults had higher concentration of tNAA than children). Additionally, a trend for group effect appeared with higher tNAA/creatine in controls than dyslexics. In the visual cortex depending on scaling and correction either only age effect or trends for group or group and age interaction were found.

**Supplementary Tables**

Table S1. Concentration of metabolites in control (CON) and dyslexic (DYS) adults and children in the left temporo-parietal cortex. Each metabolite is scaled either to water (for e.g. CHO) or creatine (for e.g. CHO/CR) and additionally adjusted for GMV by the means of linear regression.

| **Metabolite** | **No adjustment for tissue composition** | | | | | | **Adjustment for GMV** | | | | | |
| --- | --- | --- | --- | --- | --- | --- | --- | --- | --- | --- | --- | --- |
|  | **CON**  **AD** | **DYS**  **AD** | **CON**  **CH** | **DYS**  **CH** | **Main effects** | **Post-hoc** | **CON**  **AD** | **DYS**  **AD** | **CON**  **CH** | **DYS**  **CH** | **Main effects** | **Post-hoc** |
| CHO | 1.65 ±0.22 | 1.74 ±0.19 | 1.52 ±0.21 | 1.36 ±0.22 | AGE***  GR x AGE* | AD>CH  CON>DYS (CH) | 0.09 ±0.96 | 0.52 ±0.89 | -0.00 ±0.91 | -0.62 ±1.11 | AGE**  GR x AGE* | AD>CH  CON>DYS (CH) |
| CHO/CR | 0.21±0.02 | 0.22 ±0.02 | 0.24 ±0.03 | 0.22 ±0.03 | GR x AGE** | CON>DYS (CH) | -0.59 ±0.65 | -0.18 ±0.84 | 0.63 ±0.95 | -0.08 ±0.92 | AGE**  GR x AGE** | AD>CH  CON>DYS (CH) |
| GLU | 6.70 ±0.98 | 6.42 ±0.88 | 6.95 ±1.33 | 6.83 ±1.58 | AGE*** | CH>AD | 0.12 ±0.84 | -0.14 ±0.71 | 0.12 ±1.12 | 0.01 ±1.32 | - |  |
| GLU/CR | 0.87 ±0.07 | 0.82 ±0.09 | 1.14 ±0.19 | 1.05 ±0.13 | AGE***  GR* | CH>AD  CON>DYS | -0.32 ±0.39 | -0.59 ±0.46 | 0.69 ±1.06 | 0.22 ±0.72 | AGE***  GR* | CH>AD  CON>DYS |
| GLX | 8.36 ±1.19 | 7.98 ±1.00 | 8.62 ±1.47 | 8.32 ±1.67 | AGE*** | CH>AD | 0.17 ±0.92 | -0.13 ±0.74 | 0.17 ±1.13 | -0.07 ±1.26 | - |  |
| GLX/CR | 1.08 ±0.07 | 1.02 ±0.09 | 1.40 ±0.23 | 1.29 ±0.14 | AGE***  GR* | CH>AD  CON>DYS | -0.33 ±0.37 | -0.64 ±0.46 | 0.73 ±1.13 | 0.24 ±0.71 | AGE***  GR* | CH>AD  CON>DYS |
| GABA | 2.02 ±0.13 | 2.06 ±0.17 | 2.19 ±0.65 | 2.13 ±0.75 | - |  | -0.11 ±0.27 | -0.08 ±0.33 | 0.24 ±1.26 | 0.11 ±1.45 | - |  |
| GABA /CR | 0.09 ±0.00 | -0.09 ±0.01 | 0.12 ±0.03 | 0.11 ±0.04 | AGE** | CH>AD | -0.31 ±0.18 | -0.16 ±0.36 | 0.39 ±1.23 | 0.16 ±1.41 | AGE* | CH>AD |
| tNAA | 11.64 ±1.43 | 11.50 ±1.23 | 10.62 ±1.41 | 10.15 ±1.27 | AGE*** | AD>CH | 0.31 ±1.03 | 0.24 ±0.97 | -0.04 ±1.07 | -0.36 ±0.98 | AGE* | AD>CH |
| tNAA/CR | 1.51 ±0.09 | 1.49 ±0.13 | 1.69 ±0.22 | 1.58 ±0.16 | AGE**  GR*^t^* | CH>AD  CON>DYS | -0.30 ±0.48 | -0.39 ±0.67 | 0.59 ±1.12 | -0.003 ±0.82 | AGE**  GR*^t^* | CH>AD  CON>DYS |

AD - adults; CH - children; ***p<0.001; **p<0.01; *p<0.05; *^t^*p<0.1

Table S2. Concentration of metabolites in control and dyslexic adults and children in the visual cortex. Each metabolite is scaled either to water (for e.g. CHO) or creatine (for e.g. CHO/CR) and additionally adjusted for GMV by the means of linear regression.

| **Metabolite** | **No adjustment for tissue composition** | | | | | | **Adjustment for GMV** | | | | | |
| --- | --- | --- | --- | --- | --- | --- | --- | --- | --- | --- | --- | --- |
|  | **CON**  **AD** | **DYS**  **AD** | **CON**  **CH** | **DYS**  **CH** | **Main effects** | **Post-hoc** | **CON**  **AD** | **DYS**  **AD** | **CON**  **CH** | **DYS**  **CH** | **Main effects** | **Post-hoc** |
| CHO | 1.18 ±0.09 | 1.28 ±0.08 | 1.16 ±0.12 | 1.11 ±0.10 | AGE***  GR x AGE** | AD>CH  DYS>CON (AD) | -0.15 ±0.85 | 0.53 ±0.75 | 0.04 ±1.12 | -0.29 ±0.92 | GR  x AGE* | DYS>CON (AD) |
| CHO/CR | 0.15 ±0.01 | 0.16 ±0.01 | 0.15 ±0.01 | 0.15 ±0.01 | AGE**  GR x AGE** | AD>CH  DYS>CON (AD) | -0.29 ±1.02 | 0.38 ±1.27 | 0.12 ±0.69 | -0.17 ±0.97 | GR x AGE* | DYS>CON (AD) |
| GLU | 5.87 ±0.31 | 5.71 ±0.31 | 7.09 ±1.13 | 7.14 ±0.48 | AGE*** | CH>AD | -0.44 ±0.37 | -0.26 ±0.39 | 0.37 ±1.36 | 0.26 ±0.78 | AGE** | CH>AD |
| GLU/CR | 0.75 ±0.03 | 0.72 ±0.06 | 0.94 ±0.09 | 0.95 ±0.07 | AGE** | CH>AD | -0.59 ±0.63 | -0.28 ±0.53 | 0.40 ±0.84 | 0.38 ±0.87 | AGE*** | CH>AD |
| GLX | 7.79 ±0.34 | 7.52 ±0.35 | 9.01 ±1.33 | 8.95 ±0.56 | AGE*** | CH>AD | -0.34 ±0.38 | -0.28 ±0.43 | 0.38 ±1.39 | 0.16 ±0.78 | AGE** | CH>AD |
| GLX/CR | 0.99±0.04 | 0.95 ±0.08 | 1.19 ±0.11 | 1.19 ±0.09 | AGE** | CH>AD | -0.44 ±0.74 | -0.27 ±0.68 | 0.40 ±0.74 | 0.28 ±0.89 | AGE*** | CH>AD |
| GABA | 2.14 ±0.18 | 2.17 ±0.17 | 2.21 ±0.56 | 2.04 ±0.31 | - |  | 0.01 ±0.48 | 0.11 ±0.45 | 0.18 ±1.49 | -0.28 ±0.83 | - |  |
| GABA /CR | 0.11 ±0.01 | 0.11 ±0.01 | 0.12 ±0.02 | 0.11 ±0.02 | - |  | -0.06 ±0.45 | -0.01 ±0.31 | 0.27 ±1.39 | -0.21 ±1.05 | - |  |
| tNAA | 11.78 ±0.44 | 11.79 ±0.57 | 12.02 ±1.18 | 11.43 ±0.44 | GR x AGE*^t^* | CON>DYS (CH) | -0.001 ±0.56 | -0.01 ±0.72 | 0.33 ±1.49 | -0.40 ±0.55 | GR*^t^* | CON>DYS |
| tNAA/CR | 1.52±0.05 | 1.52 ±0.11 | 1.59 ±0.07 | 1.54 ±0.07 | AGE* | CH>AD | -0.16 ±0.76 | -0.04 ±1.35 | 0.45 ±0.82 | -0.28 ±0.93 | GR x AGE*^t^* | CON>DYS (CH) |

AD - adults; CH - children; ***p<0.001; **p<0.01; *p<0.05; *^t^*p<0.1
